# Case Report: Falsely elevated PTH level in a young woman caused by immunoassay interference resulting from macro-PTH

**DOI:** 10.3389/fendo.2025.1564352

**Published:** 2025-04-11

**Authors:** Giuseppe Perruolo, Lidia Santarpia, Cristina Morelli, Domenico Rendina, Federica Mormone, Giusy Ferraro, Pietro Formisano, Francesco Oriente

**Affiliations:** ^1^ Department of Translational Medicine, University of Naples Federico II, Naples, Italy; ^2^ Department of Clinical Medicine and Surgery, Federico II University, Naples, Italy

**Keywords:** parathyroid hormone, macro-PTH, chemiluminescent immunoassay, analytical interference, polyethylene glycol

## Abstract

Elevated parathyroid hormone (PTH) levels usually suggest an underlying parathyroid disorder. However, immunoassay interference, such as macro-PTH, can result in falsely elevated readings, leading to diagnostic inaccuracy. Here, we report the case of a 27-year-old woman with persistently elevated PTH levels despite normal serum calcium, phosphate, and vitamin D. Aside from a history of nephrolithiasis, the patient was asymptomatic and exhibited no parathyroid abnormalities detected on ultrasound, sestamibi scintigraphy, or choline positron emission tomography-computed tomography (PET-CT) scans. To investigate potential immunoassay interference, a polyethylene glycol (PEG) 6000 precipitation assay was performed, which showed a decrease of PTH levels from >1200 pg/mL to approximately 40 pg/mL, corresponding to a 97% reduction. To validate the specificity of this technique, the same procedure was conducted on sera from two patients with elevated PTH levels due to known parathyroid pathologies. PTH levels decreased from 771 to 271 pg/ml and 527 to 146 pg/ml, corresponding to 65 and 72% reduction, respectively. The following results indicated that the PEG precipitation primarily affected the macro-PTH in our patient’s sample while leaving intact PTH relatively unaffected in the control group. This report suggests that unexpectedly high PTH levels in the presence of otherwise normal laboratory values and imaging results could indicate the possibility of immunoassay interference. PEG 6000 precipitation is a valuable diagnostic tool for macro-PTH detection, although further refinement of immunoassay techniques may be needed to enhance the reliability of PTH measurements in clinical practice.

## Introduction

The parathyroid hormone (PTH) plays a fundamental role in regulating calcium homeostasis by acting on specific cells of bones, kidneys, and intestines. In bone, PTH stimulates osteoclast activity, leading to bone demineralization and the release of calcium and phosphate. In the kidneys, PTH stimulates calcium reabsorption and phosphate excretion, increasing the amount of calcium-free from phosphates in the blood. Starting at the kidneys, PTH stimulates the conversion of 25-hydroxy vitamin D to its active form of 1,25-dihydroxy vitamin D, which increases calcium absorption from the intestine (1, 2).

PTH levels are usually evaluated in the presence of pathologies associated with an altered calcium metabolism, such as renal failure, osteoporosis, and hyperparathyroidism or hypoparathyroidism. The determination of PTH is often performed together with the measurement of the circulating levels of phosphate, calcium, and vitamin D. Furthermore, intraoperative parathyroid hormone monitoring is useful in patients with hyperparathyroidism undergoing surgical parathyroidectomy as it allows the surgeon to evaluate the complete resection of the hyperfunctioning parathyroid tissue during the operation (3, 4). PTH is present in circulation both as a biologically active hormone and as N-terminal fragments or fragments of the intermediate or C-terminal region, which, however, maintain a high immunogenic activity in the absence of specific activity. In general, 50% of the immunoreactive component is represented by the intact hormone, the rest by the inactive fragments, whose detection can overestimate the true PTH concentration and may lead to diagnostic inaccuracies (5).

In addition to traditional second-generation immunometric assays that measure various PTH fragments, third-generation assays capable of detecting the entire PTH molecule (1–84) are now available (6–8). Although these assays have reduced most of the interference from inactive metabolites, several cases of cross-reactivity have been still described (9).

Tandem mass spectrometry (MS-MS) has also been used to characterize PTH fragments and to measure PTH in serum. The assay is relatively complex and involves a selective adsorption phase before the MS-MS step, but it appears to be of great interest because it discriminates different PTH fragments and distinguishes these molecules from the intact PTH (10, 11). To date, although MS-MS is less used than immunoassay, it appears promising for the future and might lead to a more accurate interpretation of parathyroid hormone in clinical practice.

Here, we report the clinical case of a young asymptomatic woman with elevated PTH levels due to an immunointerference assay.

## Case report

A 27-year-old woman was admitted for a day hospital procedure to the Department of Clinical Medicine and Surgery, Federico II University Hospital, Naples, Italy, for further diagnostic investigation following two episodes of nephrolithiasis and renal colic: the first about a year prior, the second three months before the one-day hospitalization. During these episodes, the patient needed to be hospitalized for sharp, knifing pain in the left flank region due to the presence of kidney stones, as confirmed by the renal ultrasound.

Her family history was positive for nephrolithiasis and renal colic since her mother and grandfather had been subject to them but was negative for autoimmune diseases.

Physical examination revealed an asymptomatic woman in good general health. In addition, she did not experience nausea, vomiting, weight loss, headaches, blurred vision, excessive thirst, constipation, muscle or joint pain, amenorrhea, or galactorrhea. However, laboratory measurements revealed consistently elevated serum PTH levels at 1250 pg/mL (reference range: 14-87 pg/mL) that were measured on the DiaSorin LIAISON XL chemiluminescent immunoassay (CLIA) analyzer. This result was confirmed 1 week (1050 pg/mL), 4 weeks (1070 pg/mL), and 4 months (1270 pg/mL) after the one-day hospitalization (**Table 1**). However, total calcium and phosphate, 25-hydroxy vitamin D, estimated glomerular filtration rate eGFR (CKD-EPI), alkaline phosphatase (ALP), and other biochemical parameters were within the normal range. **Table 2** illustrates the laboratory test results of the patient performed during the day at the hospital.

**Table 1:** PTH concentrations.

Parameter	1st measure (day hospital)	2nd measure (1 week after)	3rd measure (4 weeks after)	4th measure (4 months after)	Reference interval
PTH (pg/mL)	1250	1050	1070	1270	14 - 87

**Table 2:** Laboratory results.

Index	Results	Reference interval
Calcium (mg/dL)	9.0	8.6 - 10
Phosphate (mg/dL)	3.9	2.5 – 4.5
Magnesium (mg/dL)	2.03	1.6 – 2.6
Sodium (mmol/L)	138	138 – 145
Chlorine (mmol/L)	105	98 - 107
Urea (mg/dL)	31	16.6 – 48.5
Creatinine (mg/dL)	0.66	0.51 – 0.95
eGFR (CKD-EPI) (mL/min/1.73mq)	121	> 90
Uric acid (mg/dL)	3.0	2.4 – 5.7
AST (U/L)	17	10 - 35
ALT (U/L)	12	10 - 35
GGT (U/L)	12	6 - 42
ALP (U/L)	38	35 - 104
CK (U/L)	56	20 - 180
25-hydroxy vitamin D (ng/mL)	25	> 20
FSH (mIU/mL)	3.3	< 15
Prolactin (ng/mL)	19.2	2.8 – 29.2
TSH (μU/mL)	1.72	0.55 – 4.78
FT3 (pg/mL)	3.6	2.3 – 4.2
FT4 (ng/dL)	0.95	0.89 – 1.76
Rheumatoid factor (IU/mL)	9.75	0 - 15
IgG (g/L)	13.5	7 - 16
IgA (g/L)	2.05	0.7 – 4.0
IgM (g/L)	1.12	0.4 – 2.3

Ultrasound examination revealed a thyroid gland of normal size, shape, and position. However, a small hypoechoic, partially calcified, non-vascularized extrathyroidal nodulation was detected, leading to the hypothesis of possible parathyroid hypertrophy. Thus, the patient underwent technetium-99m sestamibi scintigraphy and then a PET-CT scan, which excluded parathyroid pathologies.

These results have led us to suspect a possible immunoassay interference. Polyethylene glycol (PEG) 6000 precipitation was performed to check this issue. In detail, PEG 6000 was diluted to 24%, mixed 1:1 with the sample serum, and incubated at 37°C for 10 minutes. The mixture was then centrifuged at 4000 rpm for 20 minutes. Finally, the supernatant was used to investigate the presence of macro-PTH (12).

The first step was to dilute the patient’s sample to check for analytical interference or methodological problems, such as verification of the linearity of the method. PTH value varied proportionally after dilution, excluding the possibility of analytical interference (**Table 3**). Interestingly, after pretreatment with PEG 6000, PTH levels decreased from 1235 to 40 pg/ml, corresponding to a 97% reduction in our patient. In contrast, in two positive controls with elevated PTH levels due to known hyperparathyroidism, PTH values dropped from 771 to 271 pg/ml and 527 to 146 pg/ml, corresponding to 65 and 72% reduction, respectively (**Table 4**). This result suggested that PEG 6000 precipitation predominantly targeted macro-PTH in our patient’s sample, with limited impact on the intact PTH in the control patients, and confirmed our hypothesis of immunoassay interference.

**Table 3:** PTH levels after sample dilution.

Sample Dilution	PTH (pg/mL)
1:1	1250
1:2	695
1:5	283,4
1:10	93,8

**Table 4:** PTH levels before and after PEG precipitation.

PTH (pg/mL)	Patient	Control 1	Control 2
Without PEG precipitation	1235	771	527
PEG precipitation	40	271	146

## Discussion

Chemiluminescent immunoassay technology ensures accurate and reliable results due to a wide dynamic range, strong signal intensity, high specificity, rapid signal acquisition, low reagent consumption, and shortened incubation times (13). However, polyreactive antibodies, autoantibodies, human anti-animal antibodies, interfering, and other binding proteins may react with the analytes leading to falsely elevated or falsely low analyte concentration and causing misinterpretation of laboratory results. Among the interfering substances, antibodies forming complexes with hormones can cause the formation of macromolecules that accumulate in blood and are measurable by the immunoassay (9, 14).

Mills et al. have reported that of 495 samples tested with elevated TSH, 3 (0.6%) were found to have macro-TSH. Similarly, Loh et al. have described an euthyroid subject with no symptoms of hypothyroidism despite the markedly elevated TSH concentration. After PEG TSH recovery, this hormone returned within the normal range, indicating the presence of macro-TSH.

Likewise, in several cases, the presence of macroprolactin has been confused with other causes of hyperprolactinemia (15), while in another case report, Gulbahar O. described a 33-year-old woman with no specific symptoms having falsely elevated PTH, TSH, ACTH, FSH, IGF-1, prolactin, β-human chorionic gonadotropin, and calcitonin levels that return to normality after to PEG precipitation (9).

PTH concentration is essential in diagnosing calcium and phosphate metabolism and is a fundamental tool for the etiological diagnosis of hypercalcemia and hypocalcemia. It is also indispensable in the intraoperative phase for the optimal success of parathyroidectomy interventions for hyperparathyroidism (3, 4). There are at least three forms of hyperparathyroidism, which can be classified as primary if the pathology is due to the presence of a parathyroid adenoma, secondary, in which there is a response of the parathyroids to a condition of hypocalcemia, tertiary, parathyroids may lose their capacity for self-regulation (16, 17).

PTH plasma concentration is increased in most patients with primary hyperparathyroidism and is lower than normal or close to the lower limit of the reference range in most patients with hypercalcemia of nonparathyroid origin, including neoplastic hypercalcemia. Moreover, normocalcemic primary hyperparathyroidism should be differentiated from secondary hyperparathyroidism, which is characterized by elevated PTH levels associated with persistently normal calcemia and is due to a physiologic stimulus to PTH secretion rather than autonomous parathyroid function. The main causes of secondary hyperparathyroidism include chronic kidney disease, malabsorption, vitamin D deficiency, calcium insufficiency, obesity, and medications (bisphosphonates, diuretics, lithium, estrogen replacement, and ferric carboxymaltose) (16).

Although not frequent, some cases of immunoassay interference due to the formation of macro-PTH causing falsely elevated PTH levels have been described.

Cavalier E et al. have shown that the serum of 34 of the 743 patients with high PTH levels measured by DiaSorin LIAISON chemiluminescence immunoassay presented an interference rate of 4.5% caused by heterophile and rheumatoid factor antibodies (18). In parallel, Levin O et al. reported a case of a 36-year-old woman whose values of PTH did not decline after parathyroidectomy despite having developed severe hypocalcemia. After performing murine- and goat-based immunoassays, PTH levels returned to 5 pg/mL, indicating a case of misdiagnosed tertiary hyperparathyroidism (19). In a very recent case report, McCarroll K et al. have described an 87-year-old osteoporotic woman with normal serum calcium, phosphate, and other biochemical parameters but elevated PTH levels evaluated by the Roche Cobas immunoassay that returned to a normal level after PEG precipitation test (20).

Two other case reports published between 2023 and 2024 showed falsely elevated PTH levels due to immunoassay interference in two patients affected by psoriatic arthritis (21) and chronic autoimmune hepatitis (22), respectively.

Here, we report a case of an asymptomatic young woman with previous episodes of renal colic, showing elevated serum PTH levels measured on the DiaSorin LIAISON XL CLIA analyzer despite the other parameters being normal. PEG precipitation assay returned the values to the normal range.

All this evidence suggests that PTH determination by immunoassays may be subject to interference that can depend on the different platforms or reagents.

In addition, our case report, along with others recently described, indicates that the problem of false positives in PTH measurement is a highly relevant issue for diagnostic medicine and patient health that should not be underestimated. In the last years, an increasing number of false positives in laboratory tests for PTH have been observed (20–22). This phenomenon can be attributed to several factors, including the presence of macro-PTH, which, being an immunological complex between PTH and immunoglobulins, can be detected in some standard tests. The implications of these false positives are significant. Misdiagnosis can lead to emotional distress for patients and inappropriate treatment. For example, a patient with a misdiagnosis of hyperparathyroidism may be subjected to unnecessary imaging tests or drug treatments that lead to unwanted side effects.

The problem of false positive PTH results could be, in part, overcome by extracting the analyte from the sample before testing, for example, through precipitation with PEG 6000.

Although the gold standard for identifying the presence of macromolecular isoforms is gel chromatography, as this method can distinguish the various isoforms of macromolecules (PRL, TSH, PTH, etc.) and determine their relative and representative quantity, it is expensive and slow.

In the normal routine of the endocrinological diagnostics laboratory, macromolecules are often determined by simpler, cheaper, and faster methods such as immunoprecipitation with PEG 6000. This compound is a polymer with high solubility in both water and other organic solutes and can bind to various macromolecules like macro-PTH, eliminating interferences from patient samples. PEG 6000-mediated precipitation is widely diffused in laboratory departments and used in clinical practice. Two other advantages of this method are the low cost and the fact that patients having macro-PTH can be followed up without medications. However, the PEG 6000 assay lacks clearly defined diagnostic cutoff limits for analyte recovery after precipitation.

Thus, PEG 6000 precipitation, despite remaining a valuable tool for detecting macro-PTH, should be recommended in combination with other strategies, including testing on other platforms and gel chromatography.

## Data Availability

The raw data supporting the conclusions of this article will be made available by the authors, without undue reservation.
